# The Impact of COVID-19 and Public Health Emergencies on Consumer Purchase of Scarce Products in China

**DOI:** 10.3389/fpubh.2020.617166

**Published:** 2020-12-02

**Authors:** Xiaotong Jin, Jianan Li, Wei Song, Taiyang Zhao

**Affiliations:** ^1^School of Business, Jilin University, Changchun, China; ^2^School of Philosophy and Sociology, Jilin University, Changchun, China

**Keywords:** public health emergencies, COVID-19, scarce consumption, materialism, need to belong, China, panic buying

## Abstract

**Objectives:** During public health emergencies, people often scramble to buy scarce goods, which may lead to panic behavior and cause serious negative impacts on public health management. Due to the absence of relevant research, the internal logic of this phenomenon is not clear. This study explored whether and why public health emergencies such as the COVID-19 pandemic stimulate consumers' preference for scarce products.

**Methods:** Applying the questionnaire survey method, two online surveys were conducted on the Credamo data platform in China. The first survey was launched in February and collected psychological and behavioral data from 1,548 participants. Considering the likelihood of population relocation due to the pandemic, a follow-up survey was conducted in August with 463 participants who had participated in the first survey and had not relocated to other cities between February and August. The hypotheses were tested with these data through stepwise regression analysis, bootstrapping, and robustness testing.

**Results:** Pandemic severity was found to positively affect scarce consumption behavior and the effect was found to be situational; this indicates that the impact of the pandemic on scarce consumption was only significant during the pandemic. Further, it was found that materialism plays a mediating role in the relationship between pandemic severity and scarce consumption. Finally, the need to belong was found to play a moderating role between pandemic severity and materialism.

**Conclusion:** This study findings imply that the scarce consumption behavior during public health emergencies can be reduced by decreasing materialism and increasing the need to belong. These findings may aid government leaders in managing public health emergencies.

## Introduction

The COVID-19 pandemic is a global public health emergency characterized with high infectivity, a high mortality rate, and a long incubation period. It affects people's psychology and behavior intensively. One of the most typical behavior during the COVID-19 pandemic is panic buying, which refers to the behavior of buying unusually large amounts of products based on the need of coping with public health emergency (1). Although panic buying has appeared worldwide, there is a dearth of empirical studies explaining it (2). Many scholars pointed out it is important to discuss panic buying from the perspective of psychology; loss of control, insecurity, social learning, and fear of scarcity may become the core factors that cause panic buying, providing a good inspiration for panic buying research (3, 4).

Given that panic buying is a complex behavior, with multiple psychological foundations, an accurate understanding and management of panic buying requires an in-depth analysis from different psychological perspectives (4, 5). Prior research from various social learning perspectives showed that in social existence, people sometimes measure the intensity of the crisis through the reaction of those that surround them. Faced with a crisis, they tend to behave in consistence with the behavior of others to cope with external shocks, which is described as conformity consumer behavior in the field (6). Research, from the perspective of decision-making mode, shows that in times of emergency, people will indulge in behaviors necessary for survival, which may lack rational thinking and lead to impulsive panic buying (7).

In fact, significant characteristics of scarce consumption (SC)—a preference for panic buying goods that are rare and only available in limited quantities—also have been observed. For example, Japanese residents anticipated that “paper products would be in short supply due to the pandemic.” As a result, they began to irrationally purchase toilet paper rolls, which caused their prices to skyrocket by a factor of 10, and they immediately went out of stock (8). Similarly, in the United States, “limited supply” or “sold out” signs on food, water, and cleaning products compelled residents to buy these items, resulting in shortages, and even social conflicts (9, 10). Due to scarcity of masks, many Chinese residents recklessly chose informal channels to purchase masks, resulting in increased fraud cases and flow of unsafe masks into the market, thus making pandemic prevention efforts even more difficult (11). For these cases, the purchase was due to scarcity rather than for pandemic prevention, as the purchase quantity surpassed the actual need; these behaviors caused a substantial panic, which negatively impacted public health management. Although SC has already exhibited significant characteristics and studies have shown that the perception of scarcity is closely related to panic buying behavior (3, 12), there have been limited research conducted from the perspective of scarcity, causing a lack of sufficient explanation for why a pandemic could cause a frantic pursuit of scarce products and how to ease panic behavior related to scarcity.

Current research tends to believe that perceived scarcity would motivate individuals to engage in panic buying due to psychological reactance and anticipated regret. Research based on the reactance theory found that health crises are likely to threaten or restrict people from buying products. Such signals will stimulate psychological resistance, which in turn will increase people's attention to products and cause panic buying (13, 14); those based on the anticipated regret theory indicates that people may regret not making panic purchases due to perceived scarcity, aiming to avoid this kind of uncomfortable feeling they will increase scarce consumption (15). Although these studies do show that scarcity and panic buying are inextricably linked, reactance and anticipated regret are broader theories, which means that they may also be applicable to the pursuit of scarce resources in other contexts. Therefore, it is necessary to conduct research on panic buying from a contextual perspective (5). In response, from a new theoretical perspective, we propose a research model of scarce consumption based on terror management theory, materialism theory, and need to belong theory, which is more suitable for panic-buying in the context of a public health crisis, aiming to explore whether and why the COVID-19 pandemic has stimulated SC, thereby enriching the research on panic buying and providing reference for crisis-response and public health emergency management.

## Hypothesis

### Scarce Consumption in Public Health Emergencies

The COVID-19 pandemic is a typical public health emergency—unpredictable and threatening. Furthermore, due to its sudden and unexpected occurrence, resources required to deal with this external threat could not be prepared sufficiently in time. Consequently, it triggered negative feelings such as death anxiety, insecurity, and fear concerning resource scarcity (16, 17). These are all uncomfortable feelings that people desire to reduce or compensate for through a series of defensive behaviors (18). Consumption is an important defensive behavior. Although this kind of behavior does not help solve actual dilemmas, it may help people cope with threats from a psychological perspective. For instance, people have opted for conformity consumption during the COVID-19 pandemic to obtain a sense of belonging and security from the group, thereby alleviating inner fear (6). Therefore, in public health emergencies, behavior is not only affected by the actual needs related to the emergencies, but also by the psychological need to alleviate negative feelings; previous studies have indicated that SC can alleviate negative psychology. The scarcity of an item (decreased quantity and limited access) can symbolize it as precious and even increase the perceived value of almost all available similar items, especially when they convey desirable attributes (19). Compared with other types of consumption, SC can compensate more for feelings associated with lack of resources, and even alleviate insecurity (20). Therefore, this study speculates that the COVID-19 pandemic may affect SC, which increases with the pandemic severity (PS).

***Hypothesis 1 (H1): Pandemic severity positively affects the scarce consumption behavior*.**

As SC is generated by a psychological need to alleviate negative feelings caused by a public health emergency, the effect in Hypothesis 1 is situational. Once a pandemic eases and people are less affected by external threats, they no longer desire to alleviate negative feelings through SC. Hence, the following hypothesis is proposed:

***Hypothesis 2 (H2): After the pandemic eases, the effect of pandemic severity on scarce consumption behavior will diminish*.**

### Materialism

Materialism (MA) is a value that is placed on the importance of possessing material wealth in life (21). Individuals with strong MA tend to be more self-centered and their focus in life is on the pursuit of material wealth with the intent of deriving pleasure and happiness (22). There are many causes of MA, including childhood poverty experiences (23), social learning (24), and insecurity (25). Fear of death is also a major source of MA (26). Terror management theory asserts that individuals usually activate the self-esteem defense mechanism after facing the threat of death. Possessing material wealth may be an effective way to boost self-esteem (27). By possessing material wealth, individuals can also enhance the sense of life's meaning and reduce death anxiety (24). Therefore, fear of death can stimulate MA to act as a buffer and to protect the mental health. The COVID-19 pandemic exposed people to a sudden threat of death, increasing fear, insecurity, and anxiety; to reduce these uncomfortable feelings, people increased their materialistic tendencies. The following hypothesis is proposed:

***Hypothesis 3 (H3): Pandemic severity positively affects materialism*.**

Consumption is a significant behavior where individuals obtain material resources, and those with strong MA tend to consume more to meet various inner needs (28), even excessively (29). They typically pay more attention to valuable products (30) and find it difficult to resist the enticement of valuable attributes (31) Compared with ordinary goods, scarce goods contain more economic and emotional value (19). Therefore, driven by MA, people are more likely to be enticed by scarce goods. The following hypothesis is proposed:

***Hypothesis 4 (H4): Materialism positively affects scarce consumption behavior*.**

The need to cope with threats from the COVID-19 pandemic, such as the threat of death, can stimulate MA (24, 27). When MA is stimulated, people are more eager to pursue valuable goods (30, 31); they may indicate a preference for scarce goods that contain more economic and emotional value. Therefore, the following hypothesis is proposed:

***Hypothesis 5 (H5): Materialism can play a mediating role between pandemic severity and scarce consumption behavior*.**

### Need to Belong

The terror management theory also asserts that people further cope with the threat of death by enhancing close relationships (32). Establishing and maintaining close relationships with others, seeking togetherness, intimacy, attachment, and affiliations alleviate the anxiety associated with death. Therefore, individuals demonstrate various coping mechanisms when faced with threats. To enhance security and reduce risks, individuals may increase their own resources by acquiring and possessing material wealth or obtaining resources or emotional attachments from others by seeking close relationships. The former will enhance the pursuit of wealth and status, while the latter will enhance social and altruistic tendencies (33). During the COVID-19 pandemic, people have adopted various methods to cope with the threat. Some prefer to acquire material possessions, while others seek close relationships to strengthen their unions and social support systems by increasing contact with relatives, donating, participating in social assistance, and other pursuits.

Baumeister and Leary (34) defined the need to belong (NTB) as a basic social necessity for forming and maintaining interpersonal relationships. Establishing contact with others or integrating into a group not only offers objective support, such as survival resources and group shelter, but also psychological support, such as emotional attachment and security (35). Compared with those with weak NTB, people with strong NTB generally have a stronger need to establish contact with others or integrate into groups. They are more active in enhancing social connections (36) and are more inclined to comply with social norms (37). Faced with a pandemic, people with strong NTB will more likely opt to enhance close relationships as a coping mechanism, while people with a weak NTB will more likely opt to obtain material resources that enhance MA and SC. Therefore, we hypothesize that NTB can reduce the impact of PS on materialism:

***Hypothesis 6 (H6): Need to belong can play a moderating role between pandemic severity and materialism*.**

**Figure 1:**
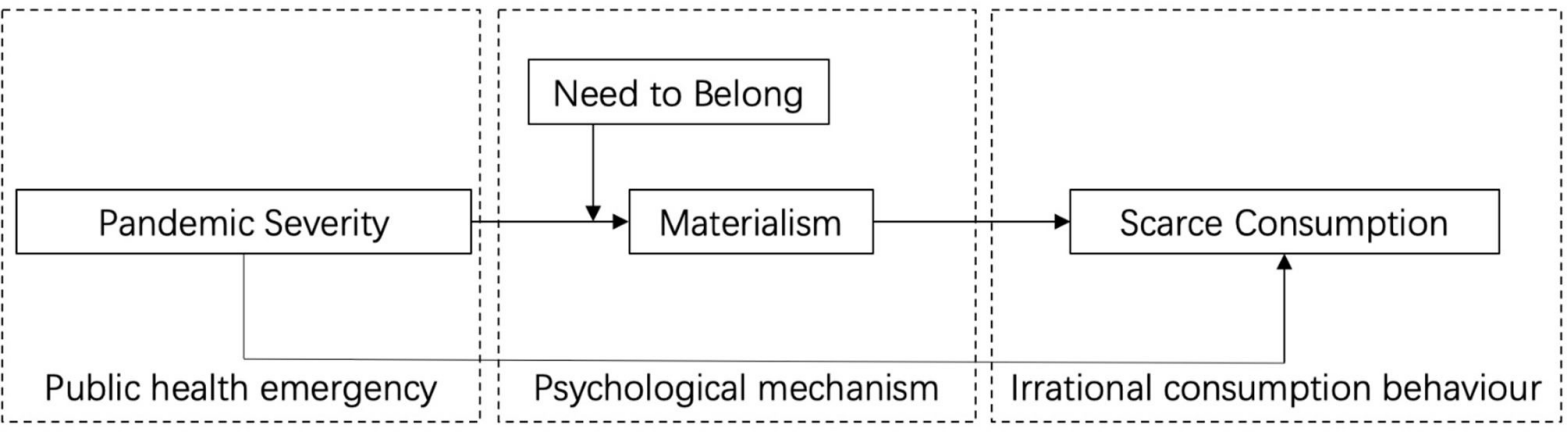
Theoretical model.

Based on above hypotheses, a psychological mechanism model for public health emergencies affecting irrational consumer behavior is proposed, as shown in Figure 1.

## Materials and Methods

### Participants and Procedure

Credamo is a professional data platform with a sample database of more than 1.5 million participants, which can provide large-scale data collection services and has been recognized by international top journals in the fields of psychology, management, sociology, and environmental science. We used it to conduct two online survey in China, one during the pandemic and one after the pandemic eased.

In the first survey, Credamo randomly distributed questionnaires in 31 provinces of China (excluding Hong Kong, Macao, and Taiwan) according to a quota of about 50 copies in each province. The survey lasted from February 15 to February 20, 2020 to covering 1,548 participants from 31 provincial-level administrative regions (excluding Hong Kong, Macao, and Taiwan) and 297 prefecture-level cities, which could accurately and comprehensively describe the psychology and behavior of Chinese citizens during the pandemic. The data was used to empirically test whether and why PS can affect SC during the COVID-19 pandemic.

To verify that the impact of PS on SC was only pertinent during the pandemic, we conducted a follow-up survey after the pandemic eased. As the COVID-19 pandemic had eased in China by August 2020, with practically no emerging cases, we distributed a follow-up survey lasting from August 3 to August 6, 2020. Considering that participants who moved location may be affected again by the pandemic severity in a new location, we used Credamo to randomly distribute the questionnaire to 500 participants who participated in the first survey, whose location had not changed between February and August. Out of these, 463 questionnaires were filled out and submitted, giving a recovery rate of 92.6%.

The questionnaire has passed the audit of Credamo, which guaranteed that it would not cause negative psychological effects on participants. As for the consent of participation, only those who agreed and volunteered to participate in surveys will access the questionnaire and corresponding remuneration. At the beginning of the questionnaire, we once again emphasized that “the survey results are only used for academic research, and the personal privacy of participants will be protected. If you agree and are participating voluntarily, start answering questions; if you disagree or are unsure, please exit.” Besides, to ensure the validity of the questionnaire, we set up test items to assess whether participants would input the answers carefully. Questionnaires that failed this test were not included in our database. Table 1 shows the demographic information of the database.

**Table 1:** Demographic information (*N* = 1,548).

**Items**	**Options**	**Sample**	**Percentage %**	**Items**	**Options**	**Sample**	**Percentage %**
Gender	Male	863	55.7	Monthly income	<3,000RMB	546	35.3
	Female	685	44.3		3,000–6,000RMB	777	50.2
Education level	High school or below	362	23.4		6,000–10,000RMB	159	10.3
	Bachelor	1,046	67.6		>10,000RMB	66	4.3
	Master or above	140	9	Monthly expenses	<1,000RMB	344	22.2
Age	<25	731	47.2		1,000–3,000RMB	551	35.6
	25–40	716	46.3		3,000–5,000RMB	465	29.5
	>40	101	6.5		>5,000RMB	197	12.7

### Measure

The PS varied in different Chinese cities; therefore, we considered measuring it using official pandemic indicators issued by the National Health Commission of the People's Republic of China for different regions, namely “the cumulative number of confirmed cases per city” and “the number of new confirmed cases per city.” Among these official indicators, “the cumulative number of confirmed cases per city” focus on the cumulative dimensions and seems to be more suitable for reflect the current situation of the pandemic than “the number of new confirmed cases per city,” which concentrate on the growth dimensions. Therefore, the former was used to reflect pandemic severity for hypothesis testing, while the latter was used for robustness testing. Therefore, we select them to measure pandemic severity, which not only meets the needs of diverse sources of indicators but is also very representative. Specifically, in the first survey, we recorded the dates when participants submitted the questionnaires and the cities where they lived. Then, we searched for the official pandemic indicators in the same timeframe and same places and included these data in the database to match the corresponding psychological and behavior data.

To measure SC, we designed a three-item scale based on the research by Sharma and Alter (20), as no SC scale in the unique context of a pandemic has been developed yet. The results were as follows: In the first and second survey, the Cronbach's alpha was 0.823 and 0.865, respectively. To measure MA, we used an eight-item scale adapted from Richins (21) and used by Okazaki et al. (38); in the first and second survey, the Cronbach's alpha was 0.859 and 0.860, respectively. To measure NTB, we used a ten-item scale adapted from Leary et al. (39); in the first and second survey, Cronbach's alpha was 0.659 and 0.798, respectively. All measurements used a 5-point Likert scale with endpoints labeled “strongly agree” and “strongly disagree.”

We also measured gender, age, education level, monthly income, monthly expenses, household size, outing frequency, and degree of social isolation. As these variables may affect SC, they were used as control variables. Among them, the gender variable was dummy coded (female = 0); income, expense, and household size were logarithmised to reduce heteroscedasticity.

## Results

### Confirmatory Factor Analysis

Considering the sample's large size, which could cause chi-square expansion, we used the Bollen-Stine bootstrapping technique (5,000 bootstrapping samples) for correction (40). The fitting indexes of the measurement model in Table 2 showed that the three-factor model had the best fitting indexes and that all indexes met the eligibility criteria. The constructs in this study are independent of each other and have good discrimination validity.

**Table 2:** Fitting indexes of competition models (*N* = 1,548).

**Model**	***χ*^**2**^**	***df***	***χ*^**2**^**/***df***	**NFI**	**IFI**	**TLI**	**CFI**	**RMSEA**
Three-factor model	161.634	132	1.225	0.983	0.997	0.996	0.997	0.012
Two-factor model 1	2,319.579	134	17.310	0.752	0.763	0.729	0.763	0.103
Two-factor model 2	2,794.010	134	20.851	0.702	0.712	0.670	0.711	0.113
Two-factor model 3	2,807.056	134	20.948	0.700	0.711	0.669	0.710	0.114
One-factor model	3,619.073	135	26.808	0.614	0.623	0.572	0.622	0.129

*Three-factor model is NTB, MA, SC. Two-factor model 1 is NTB+MA, SC. Two-factor model 2 is NTB, MA+SC. Two-factor model 3 is NTB+SC, MA. One-factor model is NTB+MA+SC*.

### Common Method Biases

The common method bias is widely used in psychology and behavioral science research to eliminate systematic errors that arise due to similarity in the data source, measurement environment, project context, and characteristics of the project itself. We used Harman's single factor test to conduct factor analysis on all variables. The variation of the unrotated first factor was 24.52%, which is less than the critical standard of 40% and less than half of the total variation (55.06%). This variation indicated that the common variance is well-controlled.

### Correlation Analysis

Pearson's correlation coefficient was used to examine potential associations between the study variables. Results showed that SC correlated significantly with PS (*r* = 0.123, *p* < 0.001), MA (*r* = 0.386, *p* < 0.001), and NTB (*r* = 0.183, *p* < 0.001). Both MA (*r* = 0.094, *p* < 0.001) and NTB (*r* = 0.060, *p* < 0.05) correlated significantly with PS, indicating potential associations between the variables.

### Hypothesis Tests

#### Analysis of Main Effect

We used the cumulative number of confirmed cases as an indicator to measure PS and built regression Models 1–2 to verify whether the main effect is established and whether it is situational. Results showed that PS had a significant positive impact on SC during the pandemic (β = 0.109, *p* < 0.001), but when the pandemic eased, PS had no significant influence on SC (β = 0.008, *p* = 0.792), indicating that PS only positively affects SC during the pandemic (see Table 3 for details).

**Table 3:** The result of stepwise regression analysis (*N* = 1,548).

**Variable**	**SC**						**MA**			
	**Model 1**	**Model 2**	**Model 3**	**Model 6**	**Model 7**	**Model 8**	**Model 4**	**Model 5**	**Model 9**	**Model 10**
Gender (female = 0)	−0.053	0.068	−0.031	−0.064^*^	−0.061^*^	−0.034	−0.056^*^	−0.064^*^	−0.085^***^	−0.082^***^
Age	−0.094^**^	−0.032	−0.042	−0.082^**^	−0.079^*^	−0.039	−0.160^***^	−0.149^***^	−0.124^***^	−0.121^***^
Education level	0.105^***^	0.136^**^	0.098^***^	0.099^***^	0.099^***^	0.097^***^	0.018	0.019	0.006	0.007
Monthly income	−0.005	0.013	0.013	−0.005	−0.009	0.009	−0.017	−0.052	−0.051	−0.055
Monthly expenses	0.127^***^	0.065	0.066^*^	0.132^***^	0.134^***^	0.073^*^	0.171^***^	0.172^***^	0.182^***^	0.184^***^
Household size	−0.053^*^	−0.037	−0.048	−0.058^*^	−0.056^*^	−0.049	−0.022	−0.015	−0.024	−0.022
Degree of social isolation	−0.041	0.034	−0.035	−0.051	−0.051	−0.039	−0.008	−0.016	−0.036	−0.037
Outing frequency	0.046	0.016	0.031	0.040	0.040	0.030	0.036	0.042	0.029	0.029
CNC	0.109^***^	0.013	0.070^**^	0.098^***^	0.095^***^	0.067^**^		0.111^***^	0.089^***^	0.086^***^
MA			0.352^***^			0.330^***^				
NTB				0.170^***^	0.171^***^	0.059^*^			0.339^***^	0.340^***^
CNC*NTB					−0.057^*^	−0.040				−0.052^*^
*R^2^*	0.061	0.061	0.037	0.089	0.093	0.182	0.048	0.059	0.172	0.174
*Adj.R^2^*	0.055	0.055	0.017	0.083	0.086	0.175	0.043	0.053	0.166	0.168
*F*	10.227^***^	1.798	30.586^***^	13.882^***^	13.106^***^	26.223^***^	8.945^***^	9.900^***^	29.325^***^	27.140^***^

* <0.05;

** <0.01;

*** *<0.001(two-tailed)*.

Therefore, H1 and H2 were verified. Pandemic severity can positively affect the scarce consumption behavior. When the pandemic becomes more serious, scarce consumption behavior will become stronger. However, the effect will diminish after the pandemic eases, indicating that it is situational.

#### Analysis of Mediating Effect

By applying the regression analysis method proposed by Baron and Kenny (41), we built Models 3–5 to test the mediating role of MA between PS and SC. Results showed that PS had a significantly positive impact on MA (β = 0.111, *p* < 0.001), which had a significantly positive impact on SC (β = 0.352, *p* < 0.001). After the addition of MA, PS had a significant impact on SC (β = 0.070, *p* < 0.01) indicating that MA played a partially mediating role between PS and SC during the pandemic (see Table 3 for details).

Following Preacher et al. (42), we used a bootstrap procedure to re-verify the mediating effect. We calculated a 95% confidence interval (CI) of the total, direct, and indirect effects through 5,000 sampling. If the CI was not zero, this mediating effect was verified as significant. Results showed that the CI of the total effect (β = 0.063, 95% CI: 0.032–0.094), indirect effect (β = 0.023, 95% CI: 0.012–0.034), and direct effect (β = 0.040, 95% CI: 0.012–0.069) were not zero, indicating that MA actually played a partial mediating role between PS and SC during the pandemic.

Therefore, H3–H5 were verified. Pandemic severity can positively affect materialism and materialism can positively affect scarce consumption behavior. Pandemic severity will affect scarce consumption behavior by affecting materialism, resulting materialism plays a mediating role between pandemic severity and scarce consumption behavior.

#### Analysis of Moderating Effect

We built regression Models 6–10 to analyze the moderating effect of NTB between PS and MA. We centralized all variables to reduce multicollinearity between them. The results showed that the interaction had a significantly negative effect on SC (β = −0.057, *p* < 0.05) and MA (β = −0.052, *p* < 0.05). However, after adding MA as a mediating variable, the effect of MA on SC was significant (β = 0.330, *p* < 0.001), but the effect of the interaction on SC was no longer significant (β = −0.040, *p* > 0.05), thus, NTB passed the test indicating that MA plays a moderating role (see Table 3 for details).

The bootstrap procedure was used to calculate the magnitude of the mediating effect and resulted in a 95% CI when the moderating variable was equal to average—one standard deviation above and one below average. The moderating effect was then re-verified through 5,000 sampling. Results showed that the interaction had a significantly negative impact on MA (β = −0.051, *p* < 0.05, 95% CI: −0.097 −0.004). Only when the value of NTB was one standard deviation higher than average was the indirect effect of PS on SC insignificant (β = 0.006, 95% CI: −0.010–0.022), indicating that NTB passed and MA plays a moderating role.

Therefore, H6 was verified. When people have a strong need to belong, the impact of pandemic severity on materialism will be reduced, resulting need to belong plays a moderating role between pandemic severity and materialism.

### Robustness Testing

We used two methods to test for and confirm the robustness of our conclusions. For the first test, we used the number of new confirmed cases per city to replace the cumulative number of confirmed cases and ensure that the effect was still stable under different indicators. For the second test, we eliminated the influence of the extreme regions in the sample, which were the most severely affected geographic area, Hubei, and the least severely affected area, Tibet. Results shown in Table 4 are consistent with the results of hypothesis testing indicating that the effect is robust.

**Table 4:** The result of robustness test (bootstrapping times = 5,000, *N* = 1,548).

**Methods**	**Effect**	**Values of NTB**	**Standardized estimate**	**S.E**.	**95% confidence interval**	
					**LLCI**	**LLCI**
Robustness test 1	Total effect	–	0.073	0.021	0.031	0.114
	Direct effect	–	0.052	0.020	0.013	0.091
	Indirect effect	–SD = 2.849	0.041	0.012	0.019	0.065
		M = 3.333	0.020	0.008	0.005	0.035
		+SD = 3.817	−0.002	0.011	−0.022	0.019
Robustness test 2	Total effect	–	0.073	0.019	0.036	0.110
	Direct effect	–	0.053	0.018	0.018	0.088
	Indirect effect	–SD = 2.849	0.024	0.009	0.008	0.042
		M = 3.333	0.015	0.006	0.004	0.027
		+SD = 3.817	0.006	0.009	−0.012	0.023

*Robustness test 1 refers to the method of replacing the CNC with the NNC; robustness test 2 refers to the method of deleting the data of “Hubei” and “Tibet”; –SD, M, +SD, respectively, represent the value of NTB is one standard deviation lower than the average, equal to average, and one standard deviation higher than the average*.

## Discussion

### Conclusions

These results indicate that pandemic severity is positively associated with materialism, need to belong, and scarce consumption. Pandemic severity positively affected the scarce consumption during the COVID-19 pandemic, and the effect was situational, which means it diminished as the pandemic eased. Materialism mediates the relationship between pandemic severity and scarce consumption. When the pandemic is severe, people demonstrate increased materialism to cope with the threat of death and, therefore, consume scarce goods. The need to belong plays a moderating role between pandemic severity and materialism, and influences people's choices in coping with death threats. People with a weak need to belong are more inclined to materialism and respond to threats by possessing scarce goods, while people with a strong need to belong prefer to cope with threats by seeking close relationships.

### Theoretical Contributions

Firstly, previous studies of public health have focused primarily on infection prediction, risk assessment, and health behaviors (16, 17, 43); however, in a public health crisis, panic behavior are inevitable for humans, and the psychological and behavioral impacts related to them also need urgent attention (44). We revealed the psychological mechanism of panic buying related to scarcity in a more in-depth way—examining whether and why public health emergencies prompt consumers to seek scarce goods—to provides a new perspective to the research of public health. Furthermore, this psychological mechanism is contextual. This makes our research different from previous studies in marketing that focused primarily on uniqueness and wealth insecurity (20, 45). We found that scarce consumption is also an important means of alleviating death threats from public health emergencies; because this research is more contextual on the basis of explaining scarcity, it is also different from those that explain scarcity based on the theory of reactance and anticipated regret (13–15).

Moreover, Arafat pointed out that panic-buying behavior may be speculatively affected by socio-cultural status, personality traits, and environmental factors, all of which needs to be empirically tested (5). We found that public health emergencies as a special social situation can stimulate materialism, while previous studies regarded it as a relatively stable personality factor (46), indicating that the psychological/behavioral constructs, linked with the personality factors, could contextually affect panic-buying behavior.

Finally, we discussed the different implications of two coping strategies in the context of pandemic. Although improving self-esteem and seeking social connections are both important coping mechanisms (32), they have different effects on mental health and public health management during a pandemic. The first approach results in materialism and scarce consumption; however, materialism is often considered as a negative social value, which negatively influences psychology and behavior leading to stinginess, jealousy, and excessive consumption (29, 47). Scarcity may activate competition orientation, leading consumers to consider their own welfare and predisposing them to act more selfishly (48) and even violently (49). Therefore, the first approach can trigger negative social behavior and hinder the management of public health. For example, according to Fox News, there were two episodes of violent conflict caused by scarce consumption of goods in the United States on March 12 alone (10). On the contrary, the second approach enhances individual social tendencies and altruistic behaviors, which can add positive and constructive significance to global health challenges (33).

### Practical Implications

The conclusions are likely to be valuable and useful to crisis-response and public-health managers. Although panic buying caused serious consequences during the pandemic, it is still easily ignored by managers. This may have been due to the belief that health needs should be given priority, rather than emergency purchases, during the COVID-19 pandemic period. Our research shows that these two goals are not conflicting and are even potentially consistent. The findings verified that the need to belong can moderate the approach witch people cope with public health emergency, thereby alleviating the panic buying related to scarcity. Moreover, the need to belong can actually increase prosocial behavior (50, 51), which is essential for the allocation of health resources in global health challenges. In other words, when people desire to obtain a sense of belonging from others or groups, they will be more inclined to think from the perspective of others or collectives. This will not only reduce the competition for scarce resources, but also increase prosocial behavior, including mutual encouragement, voluntarily helping others, even donating supplies.

In terms of specific implementation, since one of the important psychological basic for panic buying is scarcity, reducing perceived scarcity may be an effective crisis management strategy. Therefore, in order to avoid unnecessary damage to the market when fighting the pandemic, it is important for the government to inform people which products can help them cope with the pandemic and which products are unnecessary in time. It can also encourage the establishment of production, transportation and warehousing cloud platforms to realize digital management, and to realize material support more efficiently through real-time data sharing. Media should reduce the negative reports related to scarcity and replace them with healthy and positive information. To dispel rumors and reduce anxiety over shortages, business associations or organizations should cooperate with media to release information regarding production and supply; shops can adopt quota measures but try to avoid posting particularly obvious “restricted purchases” and “sold out” slogans.

On the other hand, intervention in psychological mechanisms of scarce consumption can also alleviate panic buying. The findings showed that it is effective to formulate policies or measures based on the strategy of reducing materialism and increasing need to belong. Encouraging people to increase exercising, participate in anti-pandemic topic discussions, or communicate online with relatives or friends may help to shift their attention from the material to the spiritual. In addition, public health managers should give full play to the role of the community in disseminating information, organizing activities, and distributing anti-epidemic materials, to form an atmosphere of solidarity. For example, the community can establish an information platform to provide residents with an opportunity to exchange views, while publishing material information. Further, it can encourage residents to participate in rewarded community check-in activities such as volunteer services, and regularly deliver supplies to residents isolated at home.

### Limitations and Future Research Directions

In terms of generalization of the model, this study selects China as a representative for research. Although psychological/behavioral constructs are general, they may also be affected by social-cultural factors and show certain particularities. In the future, research can focus on the comparison of different countries with different social cultures. Besides, the COVID-19 pandemic is a public health emergency with a violent and direct death threat. However, there are many types of public health emergencies, some of which pose peaceful and indirect threats. Whether these threats will lead to this kind of reactionary behavior remains to be studied.

Although exploring psychological mechanisms is important for people to understand and manage panic buying (3, 4), the influence of the media on panic buying cannot be ignored. This is because, psychological factors may in turn be influenced by the media (52) and panic buying can also be caused by unbalanced media coverage. For example, when media display the photos of empty shelves indicating the scarcity, they help to increase tension, anxiety, and fear among the general population, resulting in further increase in panic buying (2). However, this study lacks adequate consideration of the media. It did not evaluate the duration of reading or watching news. Whether there is any association between the duration of watching news and panic-buying behavior remains to be studied. In addition, people will not only be influenced by the official media but seek information from various media sources during a pandemic. Therefore, future research can also explore the impact of different media sources reporting on panic buying as well as the psychological mechanism behind it. Furthermore, we indicated that reducing the perception of scarcity and increasing the need to belong can alleviate scarce consumption behavior. But how to translate this discovery into actionable measures to a greater extent? Studies have shown that the media can spread rumors as well as health information, thus the formulation and implementation of media guidelines may help control the episodes of panic buying (52). We suggest that the combination of media and psychological guidance strategies can be studied from the perspective of policy measures in the future.

Finally, we found that after excluding the impact of social isolation, our model is still significant. This shows that although the government's isolation and restrictive policies can cause panic (53), they are not entirely responsible for causing panic, which is influenced by various factors. Future research may be carried out from the perspective of panic sources, such as the public, experts, and media, to explore the effects of these sources on panic-buying behavior, which will help to clarify the psychological mechanism of panic buying in more detail, and propose targeted strategies or policies.

## Data Availability Statement

The original contributions presented in the study are included in the article/Supplementary Materials, further inquiries can be directed to the corresponding author/s.

## Ethics Statement

The studies involving human participants were reviewed and approved by Jilin University; Credamo Data Research platform. Written informed consent for participation was not required for this study in accordance with the national legislation and the institutional requirements.

## Author Contributions

XJ, JL, and TZ were involved in the conceptualization, methodology and investigation referred to in this paper. XJ provide leadership to the team and was responsible for revising the article. JL wrote the first draft of the paper and all authors provided a written contribution and approved the final version. WS revised the manuscript. All authors agreed the final version.

## Conflict of Interest

The authors declare that the research was conducted in the absence of any commercial or financial relationships that could be construed as a potential conflict of interest.
