# Supplementary material for: The Impact of COVID-19 and Public Health Emergencies on Consumer Purchase of Scarce Products in China
Source: Front Public Health. 2020 Dec 2;8:617166. doi: 10.3389/fpubh.2020.617166 (PMC7738437; doi:10.3389/fpubh.2020.617166)
Supplement: Supplementary file 1 [file Data_Sheet_5.PDF]

Supplementary Material: Assessment Questionnaire

Objective Indicators

Severity of the pandemic (SP)

CNC: cumulative number of confirmed cases per city (for hypothesis tests).
NNC: number of new confirmed cases per city (for robustness tests).
These are two objective indicators issued by the National Health Commission of the People's Republic of China, which can be searched on the following website. http://www.nhc.gov.cn

Items of the Variables

Materialism (MA)

MA1: I admire people who own expensive homes, cars, and clothes.
MA2: I identify with famous international brands.
MA3: I like to own things that impress people.
MA4: Buying things gives me a lot of pleasure
MA5: I like a lot of luxury in my life.
MA6: I'd be happier if I could afford to buy more things.
MA7: My life would be better if I owned certain things (dropped) I don't have.
MA8: It sometimes bothers me quite a bit that I can't afford to buy all the things I'd like.

Need to belong (NTB)

NTB1: If other people don't seem to accept me, I don't let it bother me. (R)
NTB2: I try hard not to do things that will make other people avoid or reject me.
NTB3: I seldom worry about whether other people care about me. (R)
NTB4: I need to feel that there are people I can turn to in times of need.
NTB5: I want other people to accept me.
NTB6: I do not like being alone.
NTB7: Being apart from my friends for long periods of time does not bother me. (R)
NTB8: I have a strong "need to belong.
NTB9: It bothers me a great deal when I am not included in other people's plans.
NTB10: My feelings are easily hurt when I feel that others do not accept me.

Scarce consumption (SC)

SC1: Recently, I bought a product because it was about to be scarce.
SC2: Recently, I paid a little more for scarce goods.
SC3: Recently, I made more effort to obtain scarce goods, such as spending more time or using relations.

Note：(R) indicates that the item is reverse-scored.
